# Quantitative MRI assessment in metabolic dysfunctionassociated steatotic liver disease: correlation between the MRI-PDFF and liver size

**DOI:** 10.1590/0100-3984.2025.0052-en

**Published:** 2025-11-28

**Authors:** Gabriela Carboni, Louise Torres, Gabriela Furlin, Gabriela Sequeira de Campos Morais, Rubia Vanceta, Caroline Lorenzoni Almeida Ghezzi, Henrique Meira Guerra, Alice Schuch

**Affiliations:** 1 Hospital Moinhos de Vento, Porto Alegre, RS, Brazil.; 2 Hospital Cristo Redentor, Porto Alegre, RS, Brazil.; 3 Universidade do Vale do Rio dos Sinos (Unisinos), RS, Brazil.; 4 Hospital de Clínicas de Porto Alegre (HCPA), Porto Alegre, RS, Brazil.

**Keywords:** Fatty liver, Multiparametric magnetic resonance imaging, Biomarkers, Liver/diagnostic imaging, Liver/physiopathology, Fígado gorduroso, Ressonância magnética multiparamétrica, Biomarcadores, Fígado/diagnóstico por imagem, Fígado/ fisiopatologia

## Abstract

**Objective:**

To evaluate the relationship between the magnetic resonance imaging-derived proton density fat fraction (MRI-PDFF) and liver size in patients with metabolic dysfunction-associated steatotic liver disease (MASLD), as well as to explore the role of determining the craniocaudal diameter of the right hepatic lobe (CCDHL), measured at the midclavicular line, and liver volumetry as complementary tools in the assessment of hepatic steatosis.

**Materials and Methods:**

This was a single-center, cross-sectional, prospective study including 289 patients with MASLD who underwent multiparametric MRI for the evaluation of hepatic steatosis, which was categorized by the MRI-PDFF value. Liver size measurements included the CCDHL, liver volume from automated segmentation, and its difference from the total expected liver volume (eLV), calculated with the Vauthey formula.

**Results:**

A significant positive correlation was observed between the MRI-PDFF and liver size measurements, including the CCDHL (rs = 0.651; *p* < 0.001) and the eLV (rs = 0.568; *p* < 0.001). Patients with higher grades of steatosis showed a progressive increase in liver volume (*p* < 0.001). A receiver operating characteristic curve analysis demonstrated good diagnostic accuracy for the CCDHL and for the eLV in identifying moderate-to-severe steatosis (area under the curve: 0.76 and 0.83, respectively).

**Conclusion:**

The integrated assessment of the MRI-PDFF and liver size appears to be effective for the diagnosis, stratification, and monitoring of steatosis in patients with MASLD.

## Abstract (pt)

**Objetivo:**

Avaliar a relação entre a fração de gordura hepática por densidade de prótons medida por ressonância magnética (PDFF- MRI) e o tamanho do fígado em pacientes com doença hepática esteatótica associada a disfunção metabólica (MASLD). Explorar o papel do diâmetro craniocaudal do lobo hepático (DCCLH) direito medido na linha hemiclavicular e da volumetria hepática, como ferramentas complementares na avaliação da esteatose.

**Materiais e Métodos:**

Estudo transversal, unicêntrico e prospectivo incluindo 289 pacientes submetidos a ressonância magnética multiparamétrica para avaliação da esteatose hepática, a qual foi determinada pelo valor de PDFF-MRI. As medidas de tamanho do fígado incluíram DCCLH, volume hepático de segmentação automatizada e a sua diferença em relação ao volume hepático total esperado (V_oe_), calculado pela fórmula de Vauthey.

**Resultados:**

Observou-se correlação positiva significativa entre a PDFF-MRI e as medidas de tamanho hepático, incluindo o DCCLH (rs = 0,651; *p* < 0,001) e a V_oe_ (rs = 0,568; *p* < 0,001). Pacientes com maior grau de esteatose apresentaram aumento progressivo do volume hepático (*p* < 0,001). A análise da curva ROC demonstrou boa acurácia diagnóstica para o DCCLH (AUC = 0,76) e para a V_oe_ (AUC = 0,83) na identificação de esteatose moderada a acentuada.

**Conclusão:**

A avaliação integrada da PDFF-MRI e do aumento do tamanho hepático demonstrou-se eficaz para diagnóstico, estratificação e monitoramento da esteatose em pacientes com MASLD.

## INTRODUCTION

Metabolic dysfunction-associated steatotic liver disease (MASLD) is currently the most prevalent chronic liver condition worldwide, its prevalence having increased because of higher rates of obesity and type 2 diabetes. The prevalence of MASLD is approximately 38% worldwide and 44% in Latin America^**(1,2)**^. A progressive increase in its incidence has been observed in nearly all countries.

The histopathology of MASLD ranges from isolated steatosis to steatohepatitis associated with metabolic dysfunction (MASH), which can progress to fibrosis, cirrhosis, and hepatocellular carcinoma^**(1)**^. In addition to hepatic complications, MASLD is associated with an increased risk of cardiovascular, cerebrovascular, and endocrinemetabolic diseases, as well as of extrahepatic neoplasms^**(2)**^.

Early diagnosis is essential to prevent the progression of MASLD. Although liver biopsy is considered the gold standard, it is an invasive method, subject to interobserver variability and limited by sampling, as well as not taking heterogeneous parenchymal involvement into consideration^**(3,4)**^. Therefore, noninvasive methods such as determination of the multiparametric magnetic resonance imaging-derived proton density fat fraction (MRI-PDFF) have been incorporated into clinical practice^**(5)**^.

The MRI-PDFF is a quantitative, reproducible biomarker for hepatic steatosis that is quite sensitive, even at low levels (≥ 5%) of steatosis, presenting high accuracy in differentiating among the degrees of involvement, with an area under the receiver operating characteristic (ROC) curve (AUC) greater than 90%^**(5)**^.

Automated liver volumetry represents a promising method in the structural evaluation of MASLD, offering greater accuracy, less interobserver variability, and rapid analysis of large volumes of data^**(6)**^. The difference between the segmented liver volume on MRI and the expected liver volume (eLV), as determined with the Vauthey formula^**(7)**^, is calculated to evaluate volumetric deviations.

The MRI-PDFF has also been shown to correlate with the craniocaudal diameter of the right hepatic lobe (CCDHL), measured at the midclavicular line, which is a simple, accessible, reproducible measurement^**(8)**^.

The objective of this study was to evaluate the correlation between the MRI-PDFF and liver size in patients with MASLD, exploring the role of automated volumetry, determination of the eLV, and measurement of the CCDHL, as complementary tools.

## MATERIALS AND METHODS

### Participants

This cross-sectional, single-center, prospective study was approved by the institutional research board and the local research ethics committee (Reference no. 26455019. 6.3001.5330). All participants gave written informed consent. Patients were included if they were ≥ 18 years of age and were referred for multiparametric MRI of the liver for the evaluation or monitoring of hepatic steatosis between 2020 and 2021. A total of 289 such patients were considered eligible. Patients who did not present at least one of the five cardiometabolic criteria, according to the consensus for MASLD classification^**(2,9)**^, were excluded, as were those who presented with excessive alcohol use (> 20 g/ day for women and > 30 g/day for men), those who were using steatogenic medications (e.g., amiodarone, corticosteroids, methotrexate, and tamoxifen), those previously diagnosed with other liver diseases (e.g., hemochromatosis, Wilson’s disease, and alpha-1 antitrypsin deficiency), those infected with hepatitis C, hepatitis B, or HIV, those with autoimmune hepatitis, and those who were transplant recipients. It should be noted that the exclusion of patients previously diagnosed with viral hepatitis or other liver diseases was based only on the anamnesis, without laboratory or serological confirmation. Patients in whom there was technical failure on MRI (motion or metallic artifacts) were also excluded. A total of 22 patients were excluded on the basis of these criteria. Therefore, the final sample comprised 267 patients.

Data related to patient age, sex, weight, height, and abdominal circumference were collected. For each patient, the body mass index was calculated as the weight in kilograms divided by the height in meters squared (kg/m^2^).

### Imaging protocol

The images were acquired in a 1.5-T scanner (Magnetom Aera; Siemens Healthineers, Erlangen, Germany), with an 18-channel coil. Axial and coronal single-shot T2-weighted sequences were acquired, as were axial T1weighted opposed-phase gradient-echo sequences.

The software LiverLab (Siemens Healthineers) was used, with the q-Dixon technique with six echoes, generating fat (MRI-PDFF) and iron (R2*) maps, together with automated liver volumetry.

The MRI-PDFF value was classified into degrees of steatosis^**(10,11)**^: normal, < 5.6%; mild, 5.6–16.2%; moderate, 16.3–21.6%; or severe, ≥ 21.7%.

The segmented liver volume on MRI was defined as the observed volume, and the expected volume was calculated using the Vauthey formula^**(7)**^, developed for estimating liver volume during the planning of surgical resection or liver transplantation:

$$Vauthey\;formula({\text{cm}}^{3})=-794.41+1267.28\times body\;surface\;area\;\;(m^{2})$$

The eLV was defined as the difference between the expected and observed volumes.

The CCDHL (in cm) was measured at the right midclavicular line on coronal single-shot T2-weighted sequences, with a reference value of ≤ 15 cm^**(12)**^.

Liver fibrosis was assessed by using an elastography system (Resoundant, Inc., Rochester, MN, USA), with mechanical waves transmitted via a device into the right hypochondrium, generating liver stiffness maps (in kPa) to determine to what extent the presence of fibrosis influenced the eLV value obtained. Areas of low reliability and interference were avoided. The degree of fibrosis was categorized as follows^**(13)**^: normal, if < 2.5 kPa; stage F0, or chronic inflammation, if 2.5–2.9 kPa; stage F1/F2, if 3.0–3.5 kPa; stage F2/F3, if 3.5–4.0 kPa; stage F3/F4, if 4.0–5.0 kPa; and stage F4, or cirrhosis, if > 5 kPa.

The images were analyzed by two observers, working independently: a radiology fellow specializing in abdominal imaging (fourth-year resident) and a senior radiologist (with ten years of experience in abdominal radiology). The contours generated automatically in the liver segmentation were evaluated; if correct in relation to the liver surface, the total liver volume value was considered. In the MRIPDFF evaluation, nine regions of interest were created in the liver segments and compared with the automated segmentation value. If there was agreement between these values, the result of the histogram generated by the automated segmentation was used. If there was no agreement, the MRI-PDFF value used was that obtained for the largest area of the region of interest that could be adequately measured within the liver parenchyma. All examinations were initially evaluated by the radiology fellow and subsequently re-evaluated by the senior abdominal radiologist. Quantitative measurements of MRI-PDFF, R2*, and liver stiffness were performed independently by both observers, allowing the subsequent analysis of interobserver agreement. However, the CCDHL value was obtained by consensus between the two observers during the second round of reading, with the aim of ensuring methodological standardization of this anatomical measure.

### Statistical analysis

Quantitative variables are expressed as mean and standard deviation or as median and interquartile range, according to data distribution. Categorical variables are expressed as absolute and relative frequencies.

To compare medians, the Mann-Whitney or KruskalWallis test was used, with Dunn’s test for multiple comparisons. To compare proportions, we used Pearson’s chisquare test, together with analysis of the adjusted residuals.

To evaluate the power of CCDHL and eLV in predicting the occurrence of steatosis or the development of moderate-to-severe steatosis, we performed a ROC curve analysis, calculating the AUC and the 95% confidence interval.

Associations between numerical variables were assessed by calculating Spearman’s correlation coefficient.

The level of interobserver agreement between the two evaluators—for MRI-PDFF, R2*, and kPa measurements—was assessed by calculating the intraclass correlation coefficient (ICC), with interpretation according to the Landis and Koch criteria.

The significance level adopted was 5% (*p* < 0.05). All analyses were performed with the IBM SPSS Statistics software package, version 27.0 (IBM Corp.; Armonk, NY, USA).

## RESULTS

### Clinical features

The sample consisted of 267 patients with a mean age of 52.8 years, as shown in Table 1. There was a balance between the proportion of men and women (52.8% and 47.2%, respectively). The most prevalent comorbidity was obesity (in 55.8%), followed by dyslipidemia (in 50.8%) and fasting hyperglycemia (in 54.4%), corroborating the strong association between hepatic steatosis and metabolic syndrome.

**Table 1:** Clinical characteristics of the patients in the sample.

Variable	(N = 267)
Age (years), mean ± SD	52.8 ± 12.6
Sex, n (%)	
Female	126(47.2)
Male	141 (52.8)
Comorbidities, n (%)	
Obesity	148 (55.8)
Festing hyperglycemia	136 (54.4)
Type 2 diabetes mellitus	60 (22.6)
Hypertension	121 (45.5)
Dyslipidemia	135 (50.8)

Of the patients evaluated, 21% did not meet the criteria for a diagnosis of steatosis, whereas 47.2% had mild steatosis, 15% had moderate steatosis, and 16.9% had severe steatosis. The median MRI-PDFF was 10.7% (IQR: 5.9–18.1%), suggesting a predominance of mild-to-moderate steatosis.

The mean CCDHL value was 13.8 ± 2.3 cm, with 71.2% of the patients presenting a CCDHL 15 cm and 28.8% presenting a CCDHL > 15 cm. The mean liver volume obtained by automated volumetry was 1,739 ± 457 mL, whereas the expected volume, calculated by the Vauthey formula, was 1,747 cm^3 ^± 301 cm^3^, with 46.1% of the patients presenting a liver volume greater than expected.

In the ancillary evaluations, iron overload was observed in approximately 23.4% of the sample. Regarding liver fibrosis, 208 patients (77.9%) had normal results, whereas the fibrosis was categorized as stage F0, or chronic inflammation^**(13)**^, in 23 patients (8.6%). Advanced fibrosis (stage F3/F4) was uncommon, indicating that most patients were in the early stages of fibrosis. The distribution of the fibrosis stages is detailed in Table 2.

**Table 2:** Sample distribution by hepatic and metabolic characteristics.

Variable	(N = 267)
MRI-PDFF, median (IQR)	10.7 (5.9–18.1)
Degree of steatosis, n (%)	
Normal (< 5.6%)	56 (21.0)
Mild (5.6–16.2%)	126 (47.2)
Moderate (16.3–21.6%)	40 (15.0)
Severe (≥ 21.7%)	45 (16.9)
CCDHL (cm), mean ± SD	13.8 ± 2.3
CCDHL category, n (%)	
≤ 15	190 (71.2)
> 15	77 (28.8)
Observed liver volume (mL), mean ± SD	1739 ± 457
Expected liver volume (cm^3^), mean ± SD	1747 ± 301
Liver volume greater than expected, n (%)	
No	144 (53.9)
Yes	123 (46.1)
Iron overload, n (%)	
No	203 (76.6)
Yes	62 (23.4)
Degree of fibrosis, n (%)	
Technical failure	8 (2.9)
Normal (< 2.5 kPa)	208 (77.9)
F0, or chronic inflammation (2.5–2.9 kPa)	23 (8.6)
F1/F2 (3.0–3.5 kPa)	12 (4.5)
F2/F3 (3.5–4.0 kPa)	3 (1.1)
F3/F4 (4.0–5.0 kPa)	1 (0.4)
F4 or cirrhosis (> 5 kPa)	12 (4.5)

Interobserver agreement was excellent for all of the quantitative measurements evaluated. For the MRI-PDFF value, the ICC was 0.92 (95% CI: 0.90–0.94); for the R2* value, it was 1.00 (95% CI: 1.00–1.00); and for the degree of liver stiffness, it was 0.99 (95% CI: 0.99–1.00).

### Assessment of the liver dimensions

The analysis of the data demonstrated that the median eLV was −132.2 mL (IQR: −319.5 to 51.8) among the patients with a CCDHL 15 cm, compared with 275.4 mL (IQR: 41.6 to 546.8) among those with a CCDHL > 15 cm (*p* < 0.001).

In the group with a preserved CCDHL ( 15 cm), the observed liver volume was, for the most part, smaller than expected. Conversely, in the group with an increased CCDHL (> 15 cm), the median eLV was positive, indicating that the observed liver volume was larger than expected.

**Figure 1:**
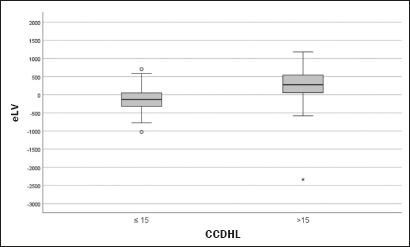
Relationship between the CCDHL and the eLV in patients with a preserved CCDHL (≤ 15 cm) and in patients with an increased CCDHL (> 15 cm).

As illustrated in Figure 1, the patients with a CCDHL > 15 cm tended to present positive discrepancies between the observed and expected liver volume, whereas those with a CCDHL 15 cm showed negative discrepancies.

**Figure 2:**
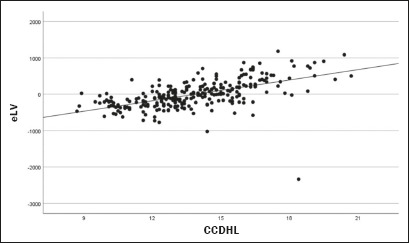
Dispersion analysis of the CCDHL and the eLV, showing a significant association between an increase in the CCDHL and an increase in the eLV.

An analysis of data dispersion (Figure 2) showed that there was a significant association between an increased CCDHL and an increased eLV, supporting the idea that the CCDHL may be a useful marker for evaluating volumetric changes in the liver in patients with MASLD.

### Correlation of sampling data

**Figure 3:**
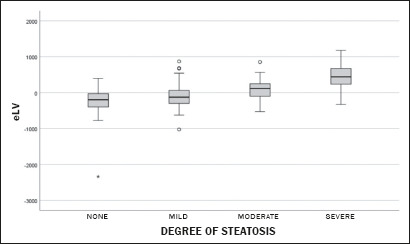
Relationship between the degree of hepatic steatosis and the eLV, indicating a direct, significant relationship between the two, especially in cases of severe steatosis.

When evaluating the relationship between the degree of steatosis presented by the patient and the eLV, we found that the eLV increased progressively with an increase in the degree of steatosis. Patients with severe steatosis had markedly larger liver volumes (441.6 mL), indicating a direct, significant relationship, as further demonstrated in Figure 3. Patients without steatosis had, on average, lower-thanexpected liver volumes, whereas those with severe steatosis had significantly higher-than-expected liver volumes. That trend was evidenced by the progression in the median values, as well as by the broader interquartile ranges in severe cases. This pattern reflects the association between hepatic fat accumulation and increased liver volume.

**Figure 4:**
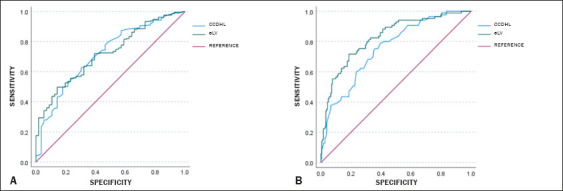
ROC curve analysis of the ability of the CCDHL and eLV to distinguish between patients with and without hepatic steatosis (A) and between moderate and severe hepatic steatosis (B).

The eLV presented an accuracy (for the presence or absence of hepatic steatosis) comparable to that of the CCDHL, with the AUC being 0.72 for both (Figure 4). However, for moderate and severe stages of the disease, the eLV had an AUC higher than that of the CCDHL (0.83 vs. 0.76), suggesting that volumetry is more sensitive for detecting the progression of steatosis.

**Figure 5:**
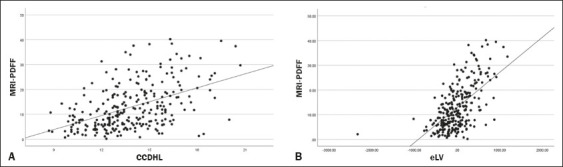
Correlations between the MRI-PDFF and the CCDHL (A) and between the MRI-PDFF and the eLV (B). Both associations were statistically significant.

**Figure 6:**
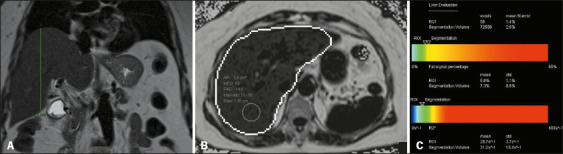
A 72-year-old female patient with diabetes mellitus, obesity, dyslipidemia, and hypertension. A: Liver of normal dimensions and contours, with a CCDHL of 12.0 cm and a volume of 1,433 mL on automated measurement. According to the Vauthey formula, the expected volume in this patient would be 1,357 cm^3^. B,C: MRI-PDFF of 5.8%, consistent with mild steatosis.

**Figure 7:**
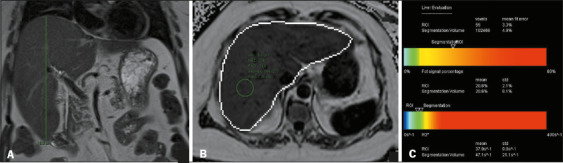
A 3-year-old female patient with diabetes mellitus, obesity, hypertension, and dyslipidemia. A: Liver with slightly blunt edges, with a CCDHL of 18.0 cm and a volume of 2,023 mL on automated measurement. The expected volume in this patient would be 1,803 cm^3^. B,C: MRI-PDFF of 20.6%, consistent with moderate steatosis.

**Figure 8:**
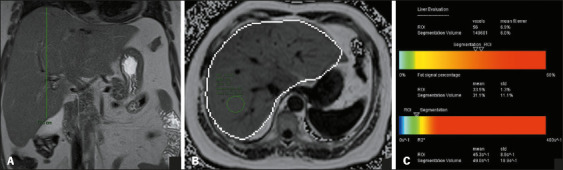
A 50-year-old female patient with obesity and hypertension. A: Liver with slightly blunt edges, with a CCDHL of 17.5 cm and a volume of 2,962 mL on automated measurement. The expected volume in this patient would be 1,778 cm^3^. B,C: MRI-PDFF of 33.5%, consistent with severe steatosis.

As depicted in Figure 5, there was a statistically significant correlation between the CCDHL and the MRIPDFF (r_s_ = 0.474; *p* < 0.001), as well as between the eLV and the MRI-PDFF (r_s_ = 0.568; *p* < 0.001), reflecting the results of the previous analyses. The interaction between fat accumulation and increased liver volume is exemplified in Figures 6, 7, and 8.

### Confounding factors

The increase in the eLV according to the degree of steatosis was not significantly different according to sex (*p* = 0.558), iron overload (*p* = 0.905) and significant degree of fibrosis (*p* = 0.315). These results highlight the role of the MRI-PDFF and liver volume in the assessment of MASLD, regardless of the influence of these confounding factors.

## DISCUSSION

### Integration of MRI-PDFF and liver size into clinical management

Our study evaluated the relationships among the MRI-PDFF, automatically segmented liver volume, and the CCDHL, aiming to better understand the interaction between fat accumulation and increased liver volume. The results demonstrate that the MRI-PDFF correlated significantly with increased liver volume, and that the eLV showed a linear progression with the degree of steatosis, being significantly higher in patients with severe steatosis. These findings support the hypothesis that steatosis induces progressive liver hypertrophy, suggesting that liver volume is an indirect marker of disease severity and a possible predictor of metabolic complications^**(3,6)**^.

The clinical relevance of these findings lies in improving MASLD assessment strategies. The use of liver volumetry automatically segmented by artificial intelligence algorithms allows an objective, reproducible estimate of liver volume, thus reducing interobserver variability and enhancing its applicability. The integration of liver volumetry and determination of the MRI-PDFF may contribute to better identification of patients at risk of progression to advanced fibrosis and metabolic complications^**(14)**^. It also raises the possibility of monitoring disease activity and assessing the therapeutic response to clinical and pharmacological interventions. Previous studies suggest that reducing the hepatic fat fraction through diet and medication is directly associated with reduced liver volume, underscoring the importance of volumetry in the monitoring of patients with MASLD^**(15)**^. Therefore, the incorporation of quantitative biomarkers, such as MRI-PDFF and liver volume, represents a significant advance in the assessment and management of the disease.

Our findings underscore the idea that MRI-PDFF is a reliable noninvasive marker for quantifying hepatic fat, with advantages over liver biopsy because it is a reproducible examination, with a larger sample volume, free from the risks associated with invasive procedures and capable of being applied at scale^**(16)**^. Our findings corroborate those of previous studies that showed liver volume to be an important factor to be considered in procedures such as liver transplantation and liver resection, given that the functional liver volume can be overestimated in the presence of steatosis^**(17)**^.

Our results are in agreement with those of Choi et al.^**(6)**^, who demonstrated a mean increase of 4.4% in liver volume for each one-point increment in the MRI-PDFF grade. In that study, the ratio of liver volume to standardized liver volume increased proportionally with the MRIPDFF grade, supporting the idea that steatosis contributes significantly to increasing liver volume. The proposal of a formula to estimate liver volume adjusted by the MRIPDFF may represent an innovative approach to correct this effect of steatosis on liver volume and improve the functional assessment of the liver^**(6)**^.

Our results are consistent with the findings of Tang et al.^**(18)**^, who demonstrated that liver volume and total hepatic fat load both showed a statistically significant correlation with the MRI-PDFF. Those authors also observed that changes in the MRI-PDFF over time were associated with changes in liver volume, underscoring the usefulness of volumetry in longitudinal disease monitoring^**(18)**^.

The heterogeneity of hepatic fat distribution poses an additional challenge to accurately quantifying the hepatic lipid load. Studies suggest that measurement of the fat fraction can lead to sampling variations and an incomplete estimate of the total hepatic lipid load. By analyzing the liver as a whole, automated volumetry minimizes these biases and allows a more accurate assessment of the lipid load^**(18)**^.

The comparison between the CCDHL and the eLV indicated that the two have similar accuracy in detecting the presence of steatosis. In addition to liver volumetry, the CCDHL has thus proven to be a relevant parameter in the assessment of liver morphology, because its measurement is a technique that is simple, widely available, and reproducible, as well as being widely applicable to different imaging techniques. The relationship between the CCDHL and the eLV observed in the present study suggests that the CCDHL can be used as an indirect marker of liver volume in patients with MASLD. In previous studies, the CCDHL demonstrated a good correlation with the presence of hepatomegaly and with the metabolic alterations associated with steatosis^**(19,20)**^. However, we found that, in the more advanced stages of steatosis, liver volume was more accurate than was the CCDHL, indicating that liver volumetry may be more sensitive in detecting steatosis progression.

In agreement with our findings, Pickhardt et al.^**(17)**^ demonstrated that total liver volume is not a good isolated predictor of fibrosis, because volumetric changes occur more through segmental redistribution than through global liver enlargement. That redistribution is reflected in the hepatic segmental volume ratio, which assesses the presence of atrophy in segments IV–VIII and compensatory hypertrophy in segments I–III^**(17)**^.

### Study limitations

Despite the relevant findings, our study has some limitations. First, this was a single-center, cross-sectional study, which limits the generalizability of the results to other populations. In addition, ethnicity, genetics, and age were not analyzed^**(19,21)**^. Furthermore, we did not evaluate anatomical variations, such as Riedel’s lobe. Moreover, the impact of heterogeneous steatosis was not taken into consideration. Other potential limitations include the absence of a longitudinal assessment, which precluded the analysis of disease progression or regression during treatment; the fact that we did not monitor patient use of medications that could impact liver volume and fat; and the fact that fasting insulin data for calculating the Homeostatic Model Assessment for Insulin Resistance (HOMA-IR) score, which is considered a secondary criterion for the diagnosis of metabolic syndrome^**(9)**^, were not available. These limitations may have reduced the representativeness of the sample and should be considered in future studies, which should include longitudinal assessments and should evaluate populations that are more heterogeneous.

### Future directions

Future studies should explore the clinical applicability of MRI-PDFF-adjusted volumetry as a predictor of metabolic and cardiovascular outcomes. The development of predictive models based on artificial intelligence may help individualize the management of MASLD, allowing therapeutic approaches to be personalized^**(16)**^. In addition, such studies should optimize automated liver segmentation and evaluate the use of eLV determination in different liver disease scenarios^**(14)**^. Another promising direction is the development of models that take lipid content into consideration in the assessment of liver function, thus informing decisions regarding surgical planning and risk stratification.

## CONCLUSION

Our data highlight the importance of integrated, noninvasive assessment of liver metrics, with emphasis on determination of the MRI-PDFF and measurement of liver volume as complementary tools in the approach to MASLD.

The MRI-PDFF stands out as a highly sensitive, accurate biomarker for quantifying liver fat, essential for risk stratification and disease monitoring. Concurrently, the assessment of liver volume, whether by automated volumetry or measurement of the CCDHL, has proven to be a metric that is practical and widely applicable, reflecting structural changes associated with the progression of steatosis.

The positive correlation between the MRI-PDFF and liver volume supports the idea that these metrics, taken together, offer a comprehensive approach to diagnosis and to the early identification of patients at higher risk of complications. In addition, the integration of these tools enables personalized therapeutic interventions, contributing to improved outcomes in patients with MASLD.

## Data Availability

The data supporting the results of this study are published in the body of this article.
